# Attrition of undergraduate nursing students at selected South African universities

**DOI:** 10.4102/curationis.v39i1.1558

**Published:** 2016-08-30

**Authors:** Erna Roos, Anna Elizabeth Fichardt, Margaret J. MacKenzie, Jacques Raubenheimer

**Affiliations:** 1 School of Nursing, University of the Free State, South Africa; 2 School of Medicine, University of the Free State, South Africa

## Abstract

**Background:**

The nursing profession forms the backbone of many healthcare systems. It therefore needs a consistent supply of registered nurses to deliver continuous and safe quality healthcare, and to replace the nurses leaving or retiring from the profession. Attrition actively occurs among nursing students in South Africa and threatens the future supply of registered nurses.

**Aim:**

The aim of the study was to describe the attrition rate at selected South African universities and the factors influencing undergraduate nursing students to discontinue their nursing studies at these universities.

**Method:**

A quantitative descriptive design was followed. Heads of the nursing departments at the selected universities captured data with a specifically designed questionnaire. Thereafter their former nursing students provided information via a structured telephonic interview on the reasons why they discontinued the nursing programme.

**Results:**

The study revealed that attrition of undergraduate nursing students for three intake years (2007, 2008 and 2009) at the participating universities was between 39.3% and 58.7%. Academic and financial reasons as well as poor wellness and health were the main causes for attrition. Another factor was failure to cope with the demands of the clinical environment.

**Conclusion:**

Attrition might not occur immediately when a nursing student is challenged, as the student might exploit the various types of support offered. Although some nursing students do benefit from the offered support, a large number of nursing students still discontinue the undergraduate nursing programme.

## Introduction

Attrition by students from academic programmes is of international concern in all higher educational settings, as all countries need an educated workforce (Angelino, Williams & Natvig 2007:2; Letseka & Maile 2008:5). Increased attrition by nursing students is of specific concern for the growth of the nursing profession and the current international shortages of nurses (Cook 2010:12; O’Holloran 2009:11). It would seem that high attrition rates from all nursing programmes is experienced in most countries, and many initiatives to improve throughput of nursing students from nursing programmes are constantly being published.

In the United Kingdom, the attrition of nursing students has been researched intensively. This is due to the critical shortage of nurses in that country and the financial burden a high attrition rate places on the taxpayer. Not only is the emphasis on the financial loss, but also on the devastating effect of attrition on the nursing students and their families (BBC news 2008:para.1). In 2006, an alarming attrition rate of 56% of nursing students was reported in the United Kingdom (BBC news 2008:para.1). By 2008, Waters (2008:20) reported an attrition rate of 26.4% in the United Kingdom. By 2011, this high attrition rate had been successfully reversed, and it was reported that a smaller number of students (8.3%) terminated their nursing programmes (Clover 2011:para.1). This was done by improved screening methods of prospective candidates, as well as supporting the nursing students through counselling, small group teaching, peer support and financial advice (Clover 2011:para.3; Waters 2008:15).

In 2009, the attrition rate among nursing students in the United States was as high as 30%. The United States as well as Jamaica and Australia reported high attrition rates that have been reduced successfully through various initiatives (Cook 2010:17; Peterson 2009:411; Stott 2007:326; Wilson 2010:para.1). Initiatives found to be successful were financial assistance through student loans, scholarships and academic assistance (Cook 2010:135; Wilson 2010:para.7).

Previous studies conducted in South Africa confirm the occurrence of attrition by nursing students. In 1995, it was reported that nursing student attrition has been an ongoing occurrence in South Africa since 1965 (Mashaba & Mhlongo 1995:371).

An attrition rate of 34.4% was reported for the 2003 intake group from a Western Cape College of Nursing (Jeptha 2008:online). It seems that a first-year nursing student was most likely to leave the nursing programme due to not reaching the programme’s minimum academic progression requirements (Mc Lachlan 2010:online). These findings were supported by research showing that the high attrition rate of first-year nursing students was mainly due to their misconception of what the nursing profession entails, and the fact that studies were offered in English, which was not the mother tongue or the daily language of many students in South Africa (Wright & Maree 2007:596).

Later research by the Solidarity Research Institute reported that only 13% of the nursing students who enrolled in 2004 in South Africa graduated in 2007. This figure reflects an alarming attrition rate as high as 87% (Solidarity 2009:online). These findings were supported by a study conducted at the Cape Peninsula University of Technology which determined that 70% of the 2005 intake of nursing students left nursing programmes within the first or second year of study (Jeptha 2008:online). A large number of these students did not really want to practise nursing, but entered the programme because they had bursaries. They were not prepared for what the clinical practice entailed and lacked direction for their future career choice (Jeptha 2008:online).

### Problem statement

From the studies mentioned above, it would seem that attrition does actively occur among nursing students in South Africa. Of concern is that no further research has been done to determine whether the trend of attrition among undergraduate nursing students has been successfully reversed. Furthermore, not many of the studies were done at higher education institutions (HEI’s).

In light of the proposed change in the registered nurse qualification, the reform plans for national health in South Africa, and the resulting increased demand for the supply of nurses, there is a need to describe attrition and the factors leading to attrition of undergraduate nursing students from university-based nursing education. The results will contribute to understanding attrition better.

## Research aim

The aim of this study was to describe the attrition rate and the influences leading to undergraduate nursing students discontinuing their nursing studies at selected South African universities.

The objectives of this study were to describe the current attrition rates of undergraduate nursing students at selected universities and factors leading to the attrition.

## Definition of key concepts

### Attrition

Attrition is defined as the departure of a student from an educational programme without successful completion or graduation (Park, Perry & Edwards 2011:39).

### Undergraduate nursing student

An undergraduate nursing student is a student enrolled for a nursing programme that leads to a baccalaureate degree in nursing at an accredited HEI.

## Contribution to the field

The scientific evidence obtained in this study may aid decision makers in improving the future throughput rate of undergraduate nursing students, and alleviate the shortage of degree-qualified nurses in the country as a whole.

## Literature review

Comparison of graduation and attrition rates is used as an important quality assurance tool within HEI’s (O’Donnell 2011:55). High attrition rates can be seen as a failure by the university to reach its primary goal (Bean n.d.:online) of assisting students in developing high and higher middle-level skills, and gaining the knowledge to practice their profession autonomously and independently after graduation (HESA 2009:6). Although higher educational assistance is available to all students, some students remain unable to graduate due to the challenges they encounter.

Attrition rates of between 35% and 44% have been reported since the 1990s by universities in America, Canada, the United Kingdom and Australia (Pocock 2012:2; Thelin 2010:online). Of concern is that, despite strategies to improve HEI quality, the attrition rate remains alarmingly high. In 2012, a feasibility study reported by the Organisation for Economic Cooperation and Development (OECD), found that an average of 31% of students entering higher education globally fail to graduate (Tremblay, Lalancette & Roseveare 2012:online). Furthermore, the time lost repeating programmes with no assurance that policies to improve quality in higher education are having the desired effect, is of concern.

High attrition rates seem to plague higher education persistently in South Africa. The graduation rate from universities does not reach the goal of 75%–80% set by the South African Department of Education (Bunting 2004:22; HESA 2009:10). In 2007, a graduation rate of 44% was reported (CHE 2007:online) and by 2009, only a 2% increase was noted (Strydom, Mentz & Kuh 2010:online). Of great alarm is the 48% attrition rate of students receiving financial assistance through the National Student Financial Aid Scheme (Wangenge-Ouma 2012:834), as well as the attrition rate of non-traditional students, which remains double that of traditional students (CHE 2007:17; HESA 2009:11; Letseka & Maile 2008:1; Wangenge-Ouma 2012:833). This has a devastating effect on provision of a much-needed diversely educated workforce to sustain the financial well-being of South Africa.

The main reasons for attrition seem to include various academic obstacles and certain non-academic-related factors (Mulholland *et al*. 2008:51; Taylor 2005:369).

Failure to succeed academically is one of the main reasons why nursing students leave an undergraduate nursing programme (O’Holloran 2009:online; Pryjmachuk, Easton & Littlewood 2008:151). Various factors including personal attributes and behavioural styles of the student, the specific learning style(s) of the student and the teaching style(s) of the educator, unpreparedness for higher education, not receiving education in the mother tongue, and clinical placement difficulties all contribute to attrition (Cameron *et al*. 2011:1373; Park *et al*. 2011:38; Wright & Maree 2007:597).

Non-academic pressure is the second main category of factors influencing a nursing student to leave the undergraduate nursing programme (Fowler & Norrie 2009:1198; Lewis 2010:59; Mc Lachlan 2010:online). These most often include lack of financial support, poor wellness and health on admission and during the programme, wrong career choice, and two demographic factors, namely age and gender (Mc Lachlan 2010:online; Pitt *et al*. 2012:906; Pryjmachuk *et al*. 2008:157). Non-traditional students are generally first-generation students and their parents are often not in a position to offer the continually needed financial support (Cameron *et al*. 2011:1373; Mashaba & Mhlongo 1995:365). Although there is currently no research indicating a relationship between physical and emotional wellness and academic performance, there are indications of the importance of wellness and health on achieving academic success (Van Lingen, Douwman & Wannenberg 2011:405). South African, Irish and the United Kingdom nursing students have reported insufficient knowledge about what the nursing profession entailed before entering the programme, and therefore made wrong career choices (Cook 2010:12; O’Donnell 2011:58; O’Holloran 2009:online; Wright & Maree 2007:597).

Studies have found that more mature students perform better academically (Pryjmachuk *et al*. 2008:157), perhaps because more mature students are often more motivated and committed to succeed due to previous experiences or failures. Gender is a strong indicator for attrition. Males tend to leave the nursing programmes twice as often as females (Mc Lachlan 2010:online; Pitt *et al*. 2012:906).

Lastly, it should be considered that most students rarely leave for a single reason, thus supporting the fact that multiple factors contribute to a student’s decision to leave (Cameron *et al*. 2011:1373).

A major challenge in higher education is to balance expanding enrolment numbers, diverse student characteristics, increasingly higher education fees, as well as the retention of students in undergraduate programmes (Angelino *et al*. 2007:2). Included in aspects of educational failure and great financial implications is an increasing pressure to produce a highly educated workforce to sustain the global economy (Bradley 2011:7; Letseka & Maile 2008:5).

## Research design and method

### Design

A descriptive quantitative design (Botma *et al*. 2010:83) was used to explore and understand the attrition rate among undergraduate nursing students at selected South African universities, and the factors that influenced the former nursing students to discontinue their studies. The attrition rate was calculated to obtain a numerical value, allowing the factors influencing nursing students to discontinue the nursing programme to be identified and then trended (Grove, Burns & Gray 2013:49).

### Populations and sampling

For this study, two study populations were of interest. The first was South African universities offering undergraduate baccalaureate degrees in nursing. The second was former students at these universities who had not completed their undergraduate nursing qualification for the course intake years 2007, 2008 and 2009. A sample was drawn from each population. Study sample 1 included five purposively selected universities. The inclusion criteria for this population was universities offering an undergraduate nursing programme leading to a baccalaureate degree in nursing, with registration with the South African Nursing Council as a graduate registered nurse. Furthermore, the universities had to be grant holders of the University-Based Nursing Education of South Africa project, funded by the Atlantic Philanthropies and ELMA Group of Foundations (UNEDSA n.d.:online). Only the five universities included in the study met these selection criteria. Non-probability convenience sampling was used to select study sample 2. The inclusion criteria were former nursing students who discontinued the undergraduate nursing programme during any of their 4 years of undergraduate study, starting from course intake years 2007, 2008 and 2009. Participants who were available telephonically were included.

### Data collection method

The data from both the study populations was gathered by means of newly designed questionnaires. Data gathered from study population 1, universities, was collected with an email-based questionnaire sent to the heads of the nursing departments. This method ensured that a wide variety of information could be gathered within a short period of time (Botma *et al*. 2010:135). The final questionnaire consisted of 15 items grouped to identify student selection criteria, determine the universities’ intake and attrition rates for a selected period, and contact information on the nursing students who discontinued the undergraduate nursing programme.

A structured telephonic interview was used to collect data from study population two, former nursing students. This was chosen to ensure a good response rate by keeping the required information short, concise and not too time-consuming (Polit & Beck 2012:265). Using a specifically designed structured questionnaire ensured that all participants heard the same questions, in the same sequential order. The questionnaire consisted of 20 close-ended items to gather general and demographic information. The year the former student started their studies, when the student discontinued the nursing programme, the social integration of the former student, funding of studies and lastly the reasons why the student discontinued their studies was investigated. Any suggestions to improve throughput rate in the educational facility were also included.

### Pilot study

The pilot studies were done at one of the selected universities, with former nursing students from this university. The specified selection criteria and the limited number of universities meeting the criteria formed the basis for this decision. Intake years prior to the ones used in the actual study were used for the pilot study. This pre-test not only confirmed the feasibility of the study, but also the appropriateness of the sampling process and methods to collect the data (Polit & Beck 2012:198). The outcome of the pilot study for study population 1, universities, revealed that an additional intake and completion year needed to be added to enlarge the data collected. The pilot study for study population 2, former nursing students, determined that the best time to phone the former students was in the evenings.

### Data collection process

An initial email was sent to sensitise and invite the heads of the nursing departments at the selected universities to voluntarily participate in the study. Written permission was obtained from all of the chosen participants. A second individualised email containing the questionnaire was then sent, allowing the heads of the nursing departments a period of three months to submit completed questionnaires to the researcher.

Once the names and telephone numbers of former nursing students were obtained from the participating universities, they were contacted telephonically to recruit them for the study. If no answer was received on the first call, two consecutive telephone calls were made to the number at different times of the day. If no answer was received at all, the participant was excluded from the study.

At the start of the telephonic interview, the aim of the study, the duration of the interview and the fact that participants could withdraw at any time from the study were explained. Participants were then given the opportunity to agree verbally to participate.

### Data analysis

The data gathered from former students were analysed using descriptive statistical techniques (Plichta & Garzon 2009:21). The techniques used to determine the attrition rate among undergraduate nursing students at selected South African universities included frequencies and percentages for categorical data, and means and standard deviations or medians and percentiles for the continuous data. All analyses were done using SAS/STAT software, Version 9.4 of the SAS System for Windows. Copyright © 2012 SAS Institute Inc.

These techniques enabled the researchers to describe the characteristics of the participants at the time of the study by means of demographic information, and to identify factors that influenced the former nursing students to discontinue their undergraduate nursing programme.

## Ethical considerations

Written consent to undertake the study was obtained from the Ethics Committee of the Faculty of Health Sciences of the University of the Free State. Two of the entered universities requested submission to their Ethics Committees, who both approved the study. The remaining universities consented, based on approval by the Ethics Committee of the University of the Free State. Respect for persons was implemented by allowing participants to decide willingly whether they wanted to participate or not. Prospective participants from both populations received information regarding the research and had the opportunity to consent or refuse in writing or verbally. The participants were allowed to withdraw at any time during the research, without having to give any reason for this decision.

### Potential hazards and benefits

The participants in this study were protected from any harm by applying solid research ethics for obtaining the necessary approval and consent before conducting the study (Botma *et al*. 2010:6). The respondents were informed that the results of this study might be published or used in presentations, and they were assured of confidentiality. Only the researcher had access to the participants’ identities. Respondents’ questionnaires were identified numerically and colour-coded by the researcher. The colour code connected each participant to a selected university. Furthermore, only the researcher had access to the questionnaires. The completed questionnaires were kept in a locked cabinet at the researcher’s office. Participants had no cost implications and none received any remuneration for participating. The main benefit of the study was to inform decision makers from participating universities why attrition occurs and at what rate.

### Reliability

Reliability refers to the consistent outcome of findings every time the research instrument is applied (Grove *et al*. 2013:45). Both questionnaires adhered to the requirements of stability and homogeneity. Allocating a set time for the participants of study population 1 to answer and return the questionnaire ensured instrument stability. The structured interviews of study population 2 were conducted within a month of receiving the names from the universities (Polit & Beck 2012:331). Homogeneity ensured that all the items on the research instrument consistently measured the same variable of interest, namely attrition (Botma *et al*. 2010:177). The information gathered on both research instruments determined an attrition rate over a set period, and illustrated the factors that influenced the former nursing students to discontinue their undergraduate nursing programme.

## Validity

In quantitative research, validity refers to the control of the research design and the instrument used to gather the information (Botma *et al*. 2010:175). Sampling bias can be a threat to internal validity (Polit & Beck 2012:247); therefore, inclusion criteria were set and adhered to. External validity is the degree to which findings can be generalised to other people or settings (Polit & Beck 2012:250). In this study, the possibilities for generalisation were enhanced by the selection criteria that were set as broadly as possible. Firstly, invited respondents from study population 1 came from different locations distributed across South Africa, representing various provinces, as well as urban and rural areas. Secondly, for study population 2, respondents from any of 4 different years of undergraduate studies, and from different locations in South Africa were included because of the location of the universities.

As a means to ensure face and content validity (Botma *et al*. 2010:175; Polit & Beck 2012:336), both questionnaires were designed after a thorough review of the literature regarding attrition from undergraduate programmes and specifically attrition from undergraduate nursing programmes (Astin 1993:6; HESA 2009:5; Mashaba & Mhlongo 1995:365; Park *et al*. 2011:38; Pryjmachuk *et al*. 2008:157; Tinto 1975:96; Wright & Maree 2007:601). The knowledge gained concerning attrition ensured pertinent questions were asked to determine the attrition rate at the universities, as well as the reasons why former students discontinued their studies. Experts in research methodology, a biostatistician, nursing science specialists and members of the ethics committee reviewed and evaluated both questionnaires.

## Results

### Attrition rates of undergraduate nursing students at selected universities

Two of the universities withdrew at a late stage during the study, without offering any reason. Responses were received from three of the five universities. One university provided limited information (and its attrition rate could not be determined), but did submit the names and telephone numbers of a large number of formers students. As only five universities met the inclusion criteria, no other universities could be invited to participate. The three participating universities were situated in different provinces, included the Northern, Central and Eastern region of South Africa, representing one rural and two urban universities.

**FIGURE 1:**
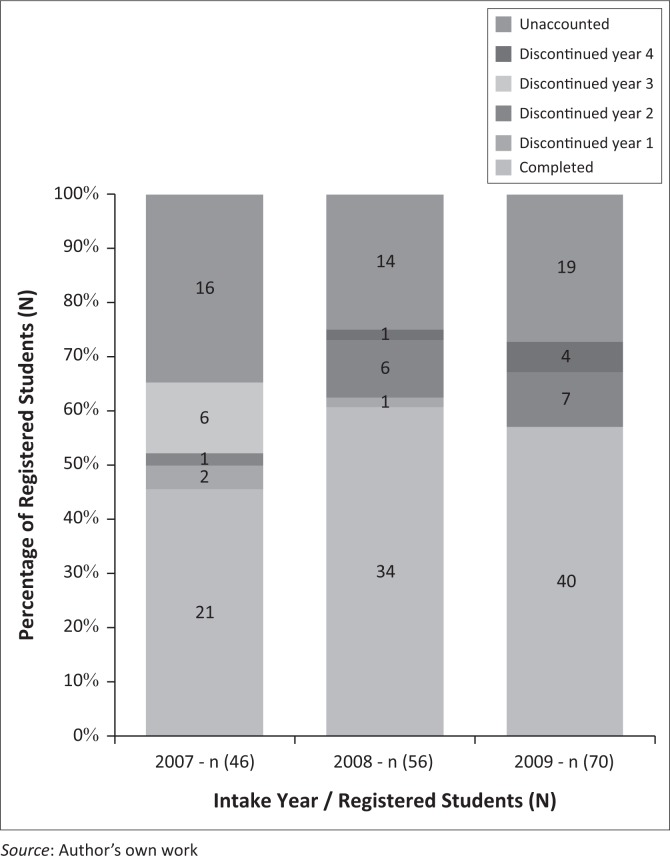
Attrition and completion rates for University one.

**FIGURE 2:**
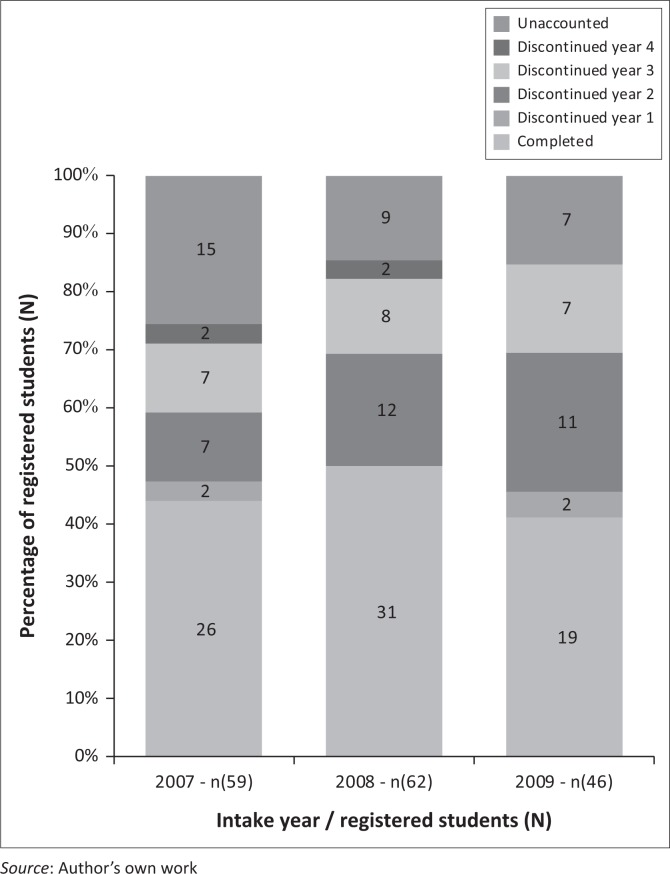
Attrition and completion rates for University two.

According to the formula on attrition by O’Holloran (2009:online), University one reported an attrition rate of 54.34% for the 2007 intake, 39.28% for the 2008 intake and 42.85% for the 2009 intake with an average of 43.49% (Figure 1). University two reported an attrition rate of 55.93% for the 2007 intake, 50% for the 2008 intake and 58.69% for the 2009 intake with an average of 54.87% (Figure 2).

### Former nursing students and factors leading to attrition

Fifty-four former students (*n* = 54) participated in the study, 3 (5.55%) from University one, 41 (75.93%) from University two, and 10 (18.52%) from University Three. Seven respondents (13%) were male and 47 (87%) were female. The ages of the students ranged from 18 to 36 years, with a mean age of 21.3 years, and a median age of 20.0 years. Twenty-three (42.60%) respondents were 19 years or younger, 10 respondents (18.52%) were 20 years old, 14 respondents (25.92%) were between 21 and 25 years old, 4 respondents (7.4%) were between 26 and 30 years old and 3 respondents (5.55%) were older than 30 years of age. The black group was the best represented with 33 respondents (61.11%), 20 respondents were white people (37.04%) and 1 (1.85%) was of mixed race. Forty-four (81.48%) respondents were single and 10 (18.52%) were married. Thirty-eight (70.37%) respondents indicated that they had no dependants during the time of their enrolment, while 16 respondents (29.63%) had dependants during the time of their study. Forty-one former students (75.93%) stayed off campus while 13 (24.07%) stayed on campus. Of the respondents staying off campus, 23 (56.10%) had their own transport and 18 (43.90%) respondents depended on public transport. Only 24 (44.44%) former nursing students ever participated in the social events arranged by the universities, with 30 (55.56%) former nursing students never participating in the social events. Most participants (61%) started their nursing course within 1 or 2 years after obtaining their National Senior Certificate.

**FIGURE 3:**
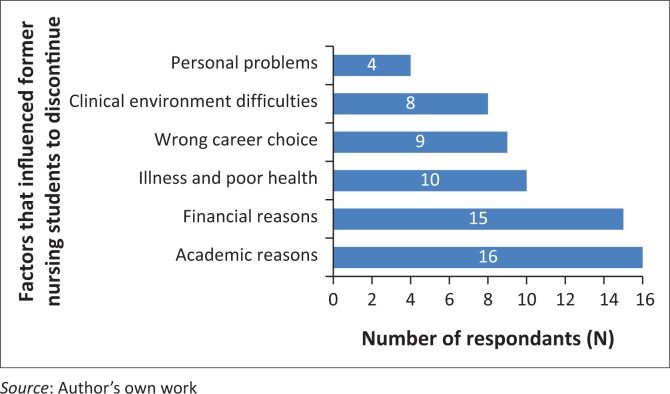
Frequency distribution of factors that influenced former nursing students to discontinued their undergraduate studies.

Thirty-five respondents (64%) discontinued the undergraduate nursing programme during their first year of study. As indicated in Figure 3, 16 (29.62%) respondents gave academic non-performance as the reason for attrition. Fifteen (27.77%) gave financial problems as a reason, while 10 (18.51%) respondents gave poor wellness and/or health problems as the reason why they discontinued their studies. Nine (16.67%) respondents reported a wrong career choice as the reason for leaving the undergraduate nursing programme. The demands of the clinical setting and their experiences in the clinical setting contributed to 8 (14.81%) of respondents’ decision to leave. Four (7.40%) respondents attributed attrition to personal problems. The total number of reasons exceeded the total number of respondents as some respondents identified more than one reason for attrition.

A majority of 29 former nursing students (53.70%) stated that no intervention could have made them continue with the undergraduate nursing programme, 12 (22.22%) respondents mentioned academic assistance, and 11 (20.37%) others believed that financial assistance would have been beneficial. Two (1.85%) respondents each stated that appropriate referrals for a health problem and information regarding what nursing entailed could have been of assistance to them.

## Discussion

The attrition rates reported by the universities were between 39.28% and 58.69%. As indicated by Figure 1, University two reported a higher attrition rate than University one. However, the attrition rate at both these universities would seem to be lower than previous reports by the Solidarity Research Institute (Solidarity 2009:online) and Cape Peninsula University of Technology (Jeptha 2008:online). Although there seem to be a decline in the attrition rates reported, of concern is that these attrition rates are significantly higher than the 20%–25% rate suggested by the Department of Higher Education in South Africa, to reach the benchmark of 75%–80% throughput rate (Bunting 2004:22; HESA 2009:10).

Existing literature indicates that year 1 and year 2 of nursing study are critical years during which attrition most often occurs (Mashaba & Mhlongo 1995:372; Mc Lachlan 2010:online). Both universities reported year 2 as the year when most students discontinued the nursing programme. University one reported one student left during year two 2 of the 2007 intake, six students left during year 2 of the 2008 intake, while seven left during year 2 of the 2009 intake. University two reported seven students left during year 2 for the 2007 intake, 12 left during year 2 of the 2008 intake, and 11 left during year 2 of the 2009 intake. As University two reported a higher attrition rate, it can be expected that a larger number of students left per year than at University one.

A surprising result obtained from University two is that many students, who had been selected to study nursing, never registered. While this is not attrition per se, it does mean that this university (and potentially the others, who did not provide information on this) started each academic year with a sub-optimal number of enrolled first-year students. These proverbial ‘empty seats’ could have been filled by other candidates.

The main factor influencing nursing students to discontinue the nursing programme was found to be academic reasons. This finding is supported by existing literature reporting academic difficulties to be the main reason why nursing students discontinue a nursing programme (Cameron *et al*. 2011:1373; O’Holloran 2009:online; Park *et al*. 2011:38; Pryjmachuk *et al*. 2008:151; Wright & Maree 2007:597). Although past academic performances are used as an indication for future academic achievements (Pitt *et al*. 2012:907), only 24 former students could remember their admission point score that was required to be selected for the nursing programme. The specific details regarding academic difficulty were not investigated in this study, and this should be further investigated in future studies.

The second reason why nursing students discontinued the nursing programme was for financial reasons. Escalating cost in higher education remains a grave concern in South Africa, making higher education unaffordable for many students, and creating difficulty for graduation (Mc Lachlan 2010:online; Wright & Maree 2007:601). Despite various forms of financial assistance available to students, only 11 of the 54 participants received a bursary.

Of interest was a third contributing factor identified as health problems, which caused discontinuation of undergraduate nursing programmes. It is not known whether the former students experienced health problems on admission or after admission to the nursing programme. Literature does suggest that nursing students who portray good physical and mental health perform better academically (Van Lingen *et al*. 2011:405).

Other factors that influenced the discontinuation of studies were identified as wrong career choice, clinical difficulties and personal problems. As suggested in the existing literature it would seem the former students were not fully informed regarding what the nursing profession entails. Once they entered the clinical setting they were disillusioned and were not able to handle the demands of what they encountered (O’Holloran 2009:online; Wright & Maree 2007:597).

### Practical implementations

The results of the study show that attrition in the undergraduate nursing programme is higher than the national average. Recommendations discussed below may contribute to improving the throughput rate of undergraduate nursing students and alleviate the shortage of degree-qualified registered nurses.

## Limitations of the study

Due to the small sample, generalisations cannot be made. However, the findings could be compared and possible trends identified.

## Recommendations

To improve the throughput rate of undergraduate nursing students at selected South African universities, the following recommendations can be suggested.

- Student selection criteria should include a personal interview and completion of health questionnaires. Personality traits and behavioural skills not portrayed on paper, as well as critical thinking skills can be determined during interviews, as well as the candidate’s actual knowledge of what the profession entails. At interview, areas of uncertainty can immediately be rectified (Schmidt & MacWilliams 2011:173). Knowledge of the current wellness and health status of a prospective student may assist in the future to refer a student for medical assistance, if necessary.
- Ensure Optimum utilisation of student admission placements by maintaining databases of the number of applicants received, number of candidates selected and number of actual student registrations. Candidates who did not register as planned may be contacted to determine the possible reason for not utilising the admission opportunity placement. The information could assist with a review on the utilisation of admission placements.
- Calculate and monitor attrition rates at nursing departments by recording current intake, attrition and completion rates. This data can be used to plan for future intakes and to determine the success of current action plans.
- Explore factors that influence the nursing student to discontinue the undergraduate nursing programme by conducting interviews with exiting nursing students. Consider the reasons given by the departing students to formulate retention strategies to improve the throughput rate from undergraduate nursing programmes. Formulate and implement retention strategies to improve the undergraduate throughput.
- Identify at-risk students for support and guidance that includes, but is not limited to academic/clinical support, financial assistance and wellness programmes.
- It is recommended that further research be done to determine the reasons why the former nursing students did not utilise the support available at the higher educational institutions. Lastly, after implementation of the above recommendations attrition rates could be studied to determine whether attrition could be managed more successfully.

## Conclusions

The findings of the study confirmed the results of previous studies on attrition. Nursing students are challenged by their own lack of persistence, an inability to adjust to the academic demands of higher education, the escalating costs to participate in higher education and a lack of integration into the social life offered at the higher educational institution as well as failure to cope with the demands of the clinical environment. Attrition might not occur immediately when a nursing student is first challenged, as the student might investigate and even exploit the various types of support on offer. Although some nursing students do find a solution when using the offered support, a large number of nursing students discontinue the undergraduate nursing programme despite the efforts.

Various retention strategies in the form of support programmes are available to the at-risk nursing student; however, despite these efforts to retain nursing students on undergraduate nursing programmes attrition occurs and will likely continue in future. Therefore, unfortunate as it is, attrition might never be diminished and might still be debated in future.
